# Brothers in arms or making a murderer? Public opinion on joint criminal enterprise

**DOI:** 10.1080/13218719.2025.2470648

**Published:** 2025-05-19

**Authors:** Simone J. Deegan

**Affiliations:** College of Business, Government and Law, Flinders University, Bedford Park, South Australia

**Keywords:** extended joint criminal enterprise, multi-defendant homicide, mandatory sentencing, public opinion, proportionality, life sentence

## Abstract

Debate continues in the academic and legal community as to the legitimacy of murder convictions based upon the doctrine of joint criminal enterprise and other forms of extended criminal liability such as ‘extended joint criminal enterprise’, ‘aiding, abetting, counselling and procuring’, and ‘constructive (or “felony”) murder’. To some extent, these cases revolve around not so much what people did but around concepts crucially related to awareness and foresight. Serious questions have been raised about such a low threshold for culpability in light of the harsh, profound, and longstanding effects that a conviction for murder automatically attracts. Drawing on focus group data, this article argues that the law of extended joint criminal enterprise is clearly at odds with community views. Moreover, current mandatory sentencing arrangements for a murder conviction under the doctrine are seen to create injustice and undermine public confidence – thereby defeating the principal rationales for mandatory sentencing in these cases.

## Introduction

An extended joint enterprise murder scenario follows. A group of young men assemble to engage in a physical confrontation with another group as an act of retribution for an earlier violent incident. A chaotic event unfolds, with altercations between individuals and small groups within the context of the broader attack. At some point, the principal offender comes across the victim and inflicts a single stab wound, killing him. A death has occurred in the commission of a multi-defendant assault, and the charge brought against every member of the aggressors’ group is murder, despite the fact they had no murderous intent at the time the victim was killed by just one person. Instead, the common law doctrine of extended joint enterprise is triggered because each male was a party to assaulting one or more of the victim’s group and, in entering into that agreement, foresaw or contemplated the possibility that one of their number *might* go beyond the scope of their plan and inflict serious harm, yet they continued to participate in the joint assault with that degree of foresight (*Chan Wing-Sui v The Queen*, 1985). Vagueness and ambiguity about the definition of ‘serious harm’ are such that the term covers a wide range of injuries, from the loss of a limb, or a wound that requires extensive treatment, to a broken bone. Under the doctrine of extended joint enterprise, it is immaterial that the young men did not want that outcome or think that there was little chance of it happening: ‘as long as it is possible, they are liable’ (Hayes & Feld, 2009, p. 29).

It is a basic tenet of the criminal law that more than one person can be liable for the commission of a crime. Yet a recurring theoretical and practical challenge regarding complicity is to analyse and assess *how* each participant might be criminally liable. That is, on what basis does the prosecution contend that a particular accused party is liable for the actions of others? This question draws attention to the way the joint criminal enterprise, particularly extended joint criminal enterprise, ‘seem[s] to over-criminalise secondary parties’ (Amatrudo, 2016, p. 922) in murder cases. Although governments internationally have endorsed the legal concept as an important weapon in the war on so-called gang violence, academic writing has approached extended joint enterprise in various ways, such as analysing it as colonial artifact, as juvenile delinquency, as distributive injustice, and as a legal problem (Hulley & Young, 2025).

By this latter term, commentators have criticised the existence of extended joint enterprise as inconsistent with the criminal law’s insistence on intention as a touchstone of liability, particularly in light of the profound and longstanding effects a conviction for murder automatically attracts (Crewe et al., 2015; Deegan, 2021; Hayes & Feld, 2009; Hulley et al., 2019; Jacobson et al., 2016; Krebs, 2017; McNamara, 2014; Parsons, 2012; Richards & McNamara, 2018; Smartt, 2018; Wang, 2019; Young et al., 2020). Indeed, mandatory head sentences of life imprisonment and mandatory non-parole periods of between 10 and 25 years for murder are reported in several jurisdictions, including the United Kingdom,^1^ Australia,^2^ and Canada.^3^ Of particular concern, juvenile offenders worldwide are often subject to adult mandatory sentencing regimes for murder, regardless of whether their involvement resides at the more moderate end of seriousness. This includes the Australian state of South Australia^4^ and, more recently, Queensland.^5^ At the same time, questions have been raised about the impact of developmental aspects of the adolescent brain (particularly in executive functioning: management of competing cognitive demands; consequence analysis; increased attraction to novel and exciting experiences; impulsivity; recklessness, etc.) on the scope and breadth of that awareness and foresight (Deegan, 2021). Concerns about the ‘dragnet’ effect of extended joint enterprise and the disproportionate rate it is experienced by Black and ethnic minority men is also the subject of a developing body of international literature (see Carvalho, 2023; Hulley et al., 2019; Squires, 2016; Williams & Clarke, 2018; Young et al., 2020). Sharing the same historical common law roots as Australia, similar principles in the United States are known to result in extreme and excessively punitive outcomes for peripheral actors, including life without parole and de facto life without parole sentences. In particular, the felony murder rule is recognised as an ‘on-ramp for extreme sentencing’ of those who neither intended to kill nor participated in the killing, thus violating the principle of proportional sentencing (Ghandnoosh et al., 2022, para. 1). As with the situation in the United Kingdom, research abounds that felony murder laws in the United States have ‘adverse impacts on people of colour, young people, and women’, particularly those ‘navigating intimate partner violence at the time of the offence … [who are] criminalised for acts of survival’ (Ghandnoosh et al., 2022, para. 5).

Unsurprisingly, an extensive body of recent research exists on public opinion around sentencing and parole (Bartels et al., 2018; Fitzgerald et al., 2016; Gelb, 2011a, b; Warner et al., 2011, 2018, 2019). Of course, it is well accepted that sentencing practices should reflect public sentiment, given that public confidence in the administration of justice is essential for peace, welfare, and good government. However, there is little research on how the public perceives variations in the roles played by the various participants in cases involving extended criminal liability. The study presented in this article makes an important and novel contribution to the literature as the first to systematically examine public opinion around the legitimacy of murder convictions – and subsequent mandatory life sentences – based on extended joint criminal enterprise.^6^ Of course, without understanding public consensus surrounding such practice, there has been limited scope to hold current practices to account. The present study was therefore designed to provide a deeper understanding of what the community wants from the criminal justice system and to produce evidence and knowledge relevant to the reform of law about whether some offenders, convicted of murder under doctrines of extended criminal liability, will not merit the mandatory sentence but rather some lesser penalty. Of course, it would be wrong to say that politicians and governments could, or should, accommodate every individual sentencing demand. But, and equally, some analytical weight attaches to the fact that decidedly arbitrary, one-size-fits-all sentencing schemes do not recognise and reflect community views about the best interests of justice. In this context, this article offers an important empirical contribution to the limited scholarship surrounding this controversial doctrine.

## The doctrine of joint criminal enterprise

Before delving into public opinion on these matters, I need to say something about the common law doctrines of *joint enterprise*, *extended joint enterprise*, *aiding and abetting*, and *constructive (or felony) murder*. It is quite clearly impossible to detail every nuance pertaining to these principles here. However, this brief framework, combined with the narratives contained herein, raise serious questions about how such severe sanctions can be enacted on such a low threshold for culpability. With that qualification, I turn now to the essential elements the prosecution must establish to prove that the deceased was murdered.

Under the criminal law, murder can take several forms. In its most basic form, it is the deliberate (i.e. not accidental) and unlawful (i.e. not in self-defence) killing of a person with an intention to kill that person or to cause them grievous harm (GBH). For example, the vignette at the centre of this article revolves around a group of teenagers and young adults who entered an affray with another group with the intention to assault members of that group. It was during such an attack that the victim was – broadly speaking – bashed, kicked, and ultimately stabbed. It is an obvious yet critical point that only one of the wounds sustained by the victim proved fatal and that not all of the accused group personally inflicted that particular wound. As a matter of logic – or as a matter of the proper exercise of the term *murder* contemplated by the criminal law – it is entirely reasonable to ask, ‘How can *all* of the group be guilty of the crime of murder when one, and only one, of its members inflicted the fatal injury?’ The answer, according to the common law, is simple enough: a person may still be guilty of the crime of murder even though they did not personally strike the fatal blow. There are three pathways by which that can occur. The first is via what is called *joint criminal enterprise* (JCE). In this situation, a person will be guilty of murder if the judge or jury is satisfied beyond reasonable doubt that the accused was part of a common plan, or joint enterprise, with others to either kill or cause GBH to another person. If one of the people who was part of that common plan performed an act that caused the death of the victim, then all of those who are part of that common plan are equally responsible for the victim’s death (*McAuliffe v The Queen*, 1995).

The second pathway, *extended criminal joint criminal enterprise* (EJCE), goes further than that. A person might reach an agreement or understanding with another or others simply to assault a person, but during that assault, one of the parties goes beyond the scope of the agreement and commits a murder. In that situation, person B can still be guilty of murder if, when they agreed to commit the crime of assault, they foresaw the possibility that person A *might* kill or *might* inflict serious harm, yet B continued to participate in the joint venture with that degree of foresight (*Chan Wing-Sui v The Queen* (1985), see also *Miller v The Queen; Smith v The Queen*; *Presley v The Director of Public Prosecutions* (2016)). It does not matter if person B does not want that outcome or thinks that there is little chance of it happening, ‘as long as it is possible, they are liable’ (Hayes & Feld, 2009, p. 29). This low threshold, commentators argue, has produced a gap between criminal responsibility and moral culpability, ‘that represents over-criminalisation and risks injustice’ (Richards & McNamara, 2018, p. 72).

For our purposes here, the third and final path via which a judge or jury can travel is to consider whether the accused were accomplices *aiding and abetting* one another. This situation arises where person B neither inflicts the fatal wound nor shares an intention (or common plan) with the killer to cause death or GBH. Instead, person B aids and abets person A when person B is present at the scene of the crime, is aware of what the perpetrator is doing,^7^ and, with that knowledge, intentionally provides encouragement, assistance, or support to the killer through words and/or their presence and behaviour (*Giorgianni v The Queen,* 1985). In fact, merely lending weight to the numbers of the accused group has been held to provide sufficient assistance in group or gang violence scenarios (*Huynh v The Queen*, 2013). At the same time, a person does not withdraw from a joint enterprise simply because they have qualms or wish they had not gotten involved. The courts are clear that it is not enough to say, ‘Don’t do it’, or ‘Don’t be a fool’. To avoid liability as a participant, a person must do what reasonably can be done which, in some way, undoes or neutralises the effect of their previous encouragement or participation in the joint enterprise (*R v Sully*, 2012).

Alternatively, *constructive murder (also referred as statutory or* *‘felony’* *murder^8^)* occurs when person A and person B are engaged in a joint enterprise to commit a serious crime.^9^ If, while that offence was on foot, Person A performed an intentional act of violence and the death of the victim resulted from that intentional act of violence, person B is also guilty of murder (*Arulthilakan v The Queen*, 2003). Constructive murder is different from the first three pathways in that it is not necessary for the prosecution to establish beyond all reasonable doubt that *either* Person A or Person B had an intention to cause death or to cause GBH. Nor is it necessary for the prosecution to establish beyond all reasonable doubt that Person B contemplated that the felonious conduct was likely to result in the infliction of death or GBH (*The Queen v Ryan and Walker*, 1966). The rationale, or public policy reason, behind constructive murder is that a person who commits a serious crime, should be held responsible for the unintended consequences of that crime (*Stuart v The Queen*, 1974). Notably in the focus of the South Australian jurisdiction, the High Court recently placed limitations on this pathway to murder, ruling that the common law concept of EJCE could not be applied to the offence of constructive murder under s 12A of the *Criminal Law Consolidation Act 1935* (SA), as to do so would create a new form of ‘constructive, constructive murder’ (*Mitchell v The King*, 2023, pp. 68–71; 96, 98). However, as legal commentator Andrew Dyer (2024) has noted,

‘[Despite] how “good” the result in *Mitchell* actually was … [t]he HCA’s ruling that EJCE and the constructive murder rule cannot be combined, came at a cost. That cost was the implicit rejection of the “change of normative position justification for EJCE”, upon which the Court had hitherto relied’ (p. 145)

As Dyer continues,

Such a rejection indicates that, as the critics of EJCE have long argued, the doctrine is in fact unjustifiable in principle – and that, accordingly, EJCE is very probably causing less ‘abstract’ injustice than the HCA has yet been willing to acknowledge. The danger is that, by cleaning up some of EJCE’s worst excesses, their Honours have made it even less likely than it was previously that Parliament in New South Wales and/or South Australia will now deal with its less obvious ones. *Mitchell* might tend to create the impression that there is no more work to be done (pp.145–146).

## Courting controversy: (extended) joint enterprise and Jogee

On June 9, 2011, Paul Fyfe – a former police officer – was stabbed once through the heart at the Leicester, England, home of his girlfriend by 25-year-old Mohammed Hirsi and, despite receiving medical attention, could not be saved. It was the prosecution case that Hirsi was ‘egged on’ to strike the fatal blow by another man, 22-year-old Ameen Jogee, who was standing outside of the residence when the stabbing occurred. In 2012, Jogee was convicted of murder under joint enterprise and sentenced to life imprisonment with a minimum 20-year prison term. However, in 2016 the United Kingdom’s highest court delivered what was seen as a major victory for civil liberties, ruling that judges had repeatedly misinterpreted earlier case law on accessorial liability – in particular EJCE – and quashing Jogee’s conviction for murder (*R v Jogee*, 2016). Heralded as righting ‘a wrong turn’ in legal history, the decision was expected to result in a sharp drop in prosecutions, as well as scores of overturned convictions in the United Kingdom and across other common law nations. Seven years later, none of that has happened. In fact, a recent review conducted by the UK Centre for Crime and Justice Studies indicates the ruling has had ‘no discernible impact’ on the number of joint enterprise prosecutions and convictions (Mills et al., 2022, p. 16). Of concern, but not surprise, the data continues to show an overrepresentation of children and young adults, and of defendants of Black appearance (p. 19). In essence, then, the principles as articulated in *McAuliffe v The Queen* and *Chan Wing-Siu v The Queen* (set out above) remain good law across the common law world.^10^ These principles were affirmed in *Miller v The Queen* (2016) and again, more recently, in *Mitchell v The King* (2023).

## The present study

Data for this article are qualitative and drawn from the second phase of a larger project on public opinion towards murder sentencing in South Australia.^11^ In the first phase, a random sample of 1556 participants, aged 19–87 years old (*M*_age_ 48.57), was recruited via social media and print advertising to participate in an online survey assessing their baseline knowledge of murder-related statistics and laws regarding murder offences in South Australia.^12^ Participants were also presented with four short vignettes depicting multi-defendant murder scenarios and asked to impose an appropriate minimum sentence range on each of the individuals described. Each scenario was based on a real-life South Australian case where each of the offenders had been convicted as either principal or secondary offenders under common law doctrines of complicity. This measure assessed if there was variation in how individuals sentenced murder offenders, as opposed to the mandatory minimum non-parole period of 20 years imposed by South Australian courts. Published elsewhere (Deegan & Jane, 2025) survey results showed that while public knowledge of actual murder-related statistics and current sentencing practices is poor, desert-based, or proportional punishments are often preferred for secondary offenders, rather than the mandatory minima.

In the second phase, reported here, 101 participants (*M*_age_ 47.72) joined focus groups that explored *how* factual scenarios and individual contributions to a crime impact public opinion on sentencing for multi-defendant murder. Participation was based on expressions of interest from survey completers. Of focus group attendees, 74.5% reported having completed Year 12 and 93.1% had additional vocational training or qualifications; 50.5% identified as male, 44.6% female, and 2.0% non-binary. Most participants reported themselves as Caucasian/white (87.1%), followed by other (7.9%), Aboriginal (3.0%), and Torres Strait Islander (1.0%). This cohort reported a median weekly income of $600–$799 per week.

Twelve 90-minute focus group sessions were convened between September and October 2022. Sessions were held in central and south metropolitan Adelaide. Two focus groups were conducted via videoconferencing to facilitate participation in hard-to-reach populations, such as those living in regional/remote South Australia – the absence of these populations was a noted limitation of previous public opinion studies (e.g. Moore, 2020). In focus groups, members of the public were presented with the scenario below, based on a real South Australian JCE case, in which each defendant was convicted of murder and sentenced to life imprisonment. Group members, as representatives of the South Australian community, were then asked to assess the conduct of each offender and to give them an appropriate sentence, if any, explaining the rationale for their decision. Participants were not given any additional information about the law or outcomes from the case until the end of the session. Using a grounded theory method (Dey et al., 2007; Glaser & Strauss, 1976), all discussion was transcribed, and themes from these transcripts were used to create an analysis framework. All quotations have been anonymised.

## Focus group scenario

Max (19 years), was assaulted by a group of young men at a house party. Max left the party then visited three friends. They agreed that, together, they would drive to the party and ‘get’ the men who had assaulted Max. As they got in the car, Max put a small knife in his pocket. When they arrived at the party, Max yelled at the men and a fight broke out. Max stabbed one of the men in the chest with the knife he had brought during the scuffle. He had not returned to the party with the intention of stabbing anyone or killing anyone; just to rough them up. But he was angry when he stabbed the man. He intended to cause the man serious harm, although not to kill him. Everyone stopped fighting as soon as they realised what had happened, and an ambulance was called. Max and his friends ran back to the car and drove off. The man who was stabbed died on the way to hospital.

Robbie (aged 19 years) is Max’s closest friend. He volunteered to return to the party because he thought the men should be punished for what they did to Max. Robbie told Max to take a weapon in case any of the men arc up to fight back. Arriving at the party, Robbie grabbed a plank of wood from the front yard and ran straight up to the rival group, hitting one of its members across the back with it. During the brawl which ensued, Robbie saw Max thrust the knife towards the victim, presumably because he was losing the fight with him. As they ran back to the car, Robbie slapped Max on the back and said, ‘Nice work! I think you got him.’

Pete (aged 18 years), is also one of the three friends who went with Max to the party. When Max suggested that they should ‘get’ the men who assaulted him, Pete agreed. Pete understood that he was agreeing to go with Max and to get into a fight with the men at the party. Pete was not armed, but he saw Max put the knife in his pocket. It occurred to Pete that Max might end up stabbing one of the men, intending to cause serious harm, if the fight got out of control. However, Pete did not want that to happen and thought there was little chance of it happening. Pete was in a fistfight with another man at the time the victim was stabbed by Max. Pete did not know that someone had been stabbed until he was in Max’s car, driving way.

Sam (aged 16 years) was also in Max’s group that night. He is a friend of Pete’s and had only met Max and Robbie that night. When Max suggested that they should ‘get’ the men who assaulted him, Sam agreed to go along. Sam wanted to impress the older boys although he had no intention of joining in the fight himself. Sam was not armed with a weapon, but he saw Max put the knife in his pocket. Where Sam comes from, a lot of boys carry knives to look tough, so he wasn’t particularly concerned about this. When they got to the party, Sam stood next to Pete, and when the fighting started, he watched and yelled support for Pete but did not throw any punches. Sam did not see Max produce the knife and only learned that someone was stabbed when he got back to the car.

### Limitations

There were several limitations in this study, including overrepresentation of certain cohorts (e.g. those who were tertiary educated and those with criminal justice experience) in both phases that may limit generalisation of conclusions to the broader public. Effort was taken to ensure all participants felt comfortable to voice their opinions, but it is possible that general consensus of the room may have censored some participants’ views through social desirability. It was also impossible to detail all of the nuances pertaining to the murder cases and legal doctrines in surveys and focus groups. However, focus group participants were provided with a similar level and depth of information on complicity that is provided to jurors in South Australian murder trials. In fact, this material was adapted directly from the prosecution opening and closing addresses that was used in these cases. As aforementioned, focus group participants (*n* = 101) self-selected from a much larger survey group of 1556 participants. It is possible that this may impact the subsequent quotes and generalisability of findings made. It is also noted that South Australia and New South Wales are the only remaining Australian jurisdictions in which the common law system of complicity applies. All other states and territories operate under a statutory complicity regime and have abolished the common law concept of EJCE (see Dyer, 2018). Consequently, results presented here may be limited by different sentencing regimes and operation of the EJCE concept. However, equivalent laws of complicity in criminal code jurisdictions (e.g. common purpose; aiding and abetting, counselling and procuring; constructive murder) still occur against a background of common law reasoning (see *R v Barlow* (1997) 188 CLR 1 per Kirby J^13^). At the same time, public opinion of the criminal justice system and the responsiveness of government policies to citizens’ preferences remains a central concern both nationally and internationally. With that qualification, I turn now to the findings of the study.

## Findings

### Making a murderer: public conceptions of the most serious crime

A key focus of the study was to capture how the South Australian public understand and view current sentencing arrangements for murder, as well as the elements of the offence that are required for a conviction. Participants generally talked about murder in instrumental terms, postulating such violent criminal conduct as deliberate, premeditated, goal-oriented behaviour requiring a string of decisions made on behalf of the offender.

A person with a sadistic nature stabbing, strangling or [doing] other violent behaviours which cost a victim their life. A murder is a cold, narcissistic, hideous wrongdoing which is becoming more common in Australia unfortunately (Alice, FG7).

For many people, murder was something to be attributed solely to the ‘dangerous other’, with the perceived depravity and cruelty of incidents enabling perpetrators to be constructed as ‘not like us’. This was most evident in focus groups with community members who regularly cited infamous cases and offenders such as Peter Sutcliffe, Ian Brady, Myra Hindley, Bevan Spencer Von Einem, and the killers of James Bulger, invoking particularly punitive responses. For example:

It makes me think of the two 10 year old boys who killed that other little boy (Anna, FG11).

Bevan Von Einem did horrific – absolutely horrific – injuries to Richard Kelvin, you know. No one should have to go through that (Marshall, FG11).

To sentence everyone on the basis of Peter Sutcliffe, in my view, is just not reasonable. I understand that people are incredibly distressed (Nadine, FG6).

Clearly, when thinking about ‘proper punishment’ for murder offenders, the public tends to use the most violent caricatures, with the highest levels of culpability, as their frame of reference. But what of those individuals who do not fit neatly in the ‘murderer’ category? Mitchell and Roberts (2012) have summed up this situation, writing that ‘the primordial justification for the mandatory life sentence is that murder is a uniquely serious crime and thus warrants a unique sentence’. But, as they continue, ‘not all murders are in fact uniquely serious’ (p. 504). To this extent, each member of the public has their own experience of the world. They each bring their own experiences into the focus group and use those experiences in their assessment of the scenario that they heard. Each participant was accustomed to making judgements about the actions of their fellow members in the South Australian community, and they used that experience when they came to consider the basic facts that they heard in the scenario.

In the same way, jurors use their collective experience from their lives in the community to weigh the evidence to determine what they accept as fact, what they reject, and the conclusions they are prepared to draw (Deegan & Jane, 2025). The following sections highlight the relevance of everyday life experience and common-sense responses – even for serious offences like murder – to these judgements.

### Devil in the detail: intention to kill versus intention to cause grievous bodily harm

In almost every focus group, members of the public were quick to identify ‘intention to kill’ as key to the guilt of an accused person. Most community members, however, expressed a lack of understanding about the offence of murder – specifically, the fact that ‘intention to cause grievous bodily harm’ was part of its definition – believing that where individuals had not intended to kill, they should be convicted of manslaughter instead. This theme was especially prominent given the scenario concerned a fight between two groups of young men that went beyond its projected outcomes. It was during such an attack that the victim was stabbed once in the chest and died en route to hospital. Almost all members of the community were very reluctant to characterise this killing in this scenario as a murder, even where the principal offender, Max, was concerned:

It was premeditated that they were going to assault them. It wasn’t a premeditated killing of someone. What happens in the heat of the moment could be that someone dies. … And yes, of course that’s bad. But they are two distinct things (Elton, FG10).

None of them should be charged with murder because none of them went there with the intention of killing anyone. There’s a big difference between the intention of killing someone and the intention to beat the shit out of someone and then accidentally stabbing someone and killing them. That’s the difference (Nicholas, FG11).

Most members of the community were of the view that murder should be reserved for ‘the most serious cases of unlawful killing, leaving manslaughter to capture those considered “less heinous”’ (Mitchell & Roberts, 2012, p. 5). Across the various groups, it was understood that such events must be met with legal sanction and moral censure, but like the prisoners interviewed by Hulley et al. (2019), the majority did not accept that *any* of the secondary offenders in the fight scenario were *morally* guilty of murder:

It’s more of a subsidiary role but an important role, I think, in the whole scuffle. In my view, Robbie would be deserving of punishment. But not for murder (William, FG5).

The telling thing is that when Max suggested that they should get them Pete agreed. He agreed to escalate the situation. Given that, he does deserve something but not murder (Marcus, FG7).

He has bolstered the numbers, so he played a role in what happened. I mean, if the guys didn’t have Sam, would they still have gone? Probably, ‘cause he’s a fairly minor character. But he did play a role because when they turned up [to fight], now there’s three or four of them there. … So, he has a role and he’s guilty of something (Neil, FG2).

The types of things that concerned community members or made them feel unsure about a murder conviction varied. Most concerns related to the issue of intent, as discussed above. Other community members talked about their own personal experiences as youths or as parents of teenage boys. This included, first and foremost, an understanding of how a youth who is not pursuing a murderous plan could get themselves into a nasty predicament where a life is lost, and the lives of many others (including their own) are instantly and irrevocably transformed:

A lot of children do things like this. … They’re not, they’re not classical murderers that go out there purposely for doing that. It’s just like wrong place wrong time for Max, that he got picked on, you know, had a fight and just got hot-headed and lost it. Came back and brought his mates to come back. I’ve been in scenarios like that where people have done that; they just lose their ability to do anything logical (Robin, FG10).

The concept of ‘victim precipitation’, whereby ‘a physical attack by the victim provokes the offender’s lethal attack’ (Felson & Messner, 1998, p. 406) was also seen to mitigate the actions of the principal offender to some extent. In a sense, as previously described by Fiddes (1981, p. 51), these hooliganesque events were seen as the typical killings ‘against which the more problematic cases stand out’. As observed by the following focus group participant:

For a large group of young men, having a fight is just part of your night out. You know, you go out, you get in a fight, and if you’re lucky at the end of it, you get a root [i.e., sex]. I know that sounds gross. But, in reality, they’re like bull terriers. You know how bull terriers are lovely dogs and they’re really friendly to people, but part of their world is just fighting other dogs (Nadine, FG6).

Nadine highlights the distinctive proclivity of some males, largely from lower socioeconomic ranks, to violently escalate real or imagined threats to their reputation or status, sometimes resulting in death (Deegan, 2021, 2024). To this extent, scholarly accounts of young men’s violence have remained remarkably consistent over time. Polk (1994, 1999) and others (see Block & Block, 1993; Luckenbill, 1977; Wolfgang, 1958) categorise this form of homicide as ‘fundamentally nothing more than a fatal assault – that is, a physical assault that escalated beyond the projected course of action’ (Warley, 2011, p. 6). In short, the degree of separation between an aggravated assault, an assault causing actual serious harm with intent, an attempted murder, and a completed murder can be frightfully narrow. This much is demonstrated by the High Court decision of *R v Crabbe* (1985) which held that a person is guilty of reckless murder if they commit an act knowing that it will probably cause death or GBH, and death in fact results.

### Hindsight as 20/20: the legitimacy of foresight as a basis for conviction

As illustrated by the focus group vignette scenario, unlike any other sort of case in the whole criminal calendar, EJCE refers to and requires foresight rather than intention. As such, the crux of the legal contentions surrounding joint enterprise, and other associated doctrines of accessorial liability, involves questions about the scope or breadth of that awareness or foresight. Of course, people in a group can, and often do, have very different expectations. What they expect depends very much on what they know. In line with that reasoning, across all focus groups, the majority position was that any legal response to group violence should be shifted towards issues of culpability and responsibility and away from the unhelpful notions of foresight and (purported) gang membership:

If you are a group of three or four mates who go off somewhere to do something, are you a ‘gang’? I don’t know (Sharon, FG1).

No one can predict what someone else is going to do. … The intent, the original intent, may have been a common thing, but you can never predict what some other person’s going to do in the spirit of the moment, the heat of the moment, when they lose it or whatever (Jamie, FG4).

Constructing multi-defendant events as gang-related is, of course, an immediately obvious and predictable means to justify a swift and punitive legal response in the face of gratuitous violence. This is evident in the legislative response to the death of Ben Kinsella, a killing that triggered a series of protests and became emblematic of deeper undercurrents of antisocial behaviour and knife crime in inner-city London. With reference to the ‘tougher sentences for knife killers’ sentencing reforms, the Ministry of Justice, UK (2023) reflected that:

There is a range of starting points for murder cases beginning at 15-years, with a 30-year starting point for murders involving firearms. And in 2010, following the murder of Ben Kinsella – who was stabbed to death in the street in what was believed to be a case of mistaken identity – we added a 25-year starting point for murders committed with a knife or other weapon taken to the scene with intent (p. 3).

It was considered that the risk posed to the public by knives being carried on the streets, with the intention of using them to cause harm, needed to be reflected in the sentencing framework for murder (p. 26).

Ben Kinsella’s father has also been quoted in media outlets describing the ‘necessity’ of extended joint enterprise to properly punish perpetrators of collective violence and give victims’ families the justice they deserve:

If it wasn’t for joint enterprise those that had cornered our son, watched and encouraged as he was stabbed and then did nothing to help him as he bled to death would likely be walking the streets today. In five seconds, he was stabbed 11 times, and I hold them all responsible. If anyone disagrees with joint enterprise then they need to talk to the families of the victims of gang violence. Then they might change their mind (Robertson, 2014).

However, as decades of research have highlighted, major ambiguities and difficulties in providing consistent definitions for gang affiliation and gang-related crime mean that ‘linking legal action to an individual’s perceived status may erroneously criminalise that individual’ (Esbensen et al., 2001, p. 125). Furthermore, members of the public openly questioned the relevance of the term ‘gang’ to Australian youth activity. For them, the joint enterprise legislation was created with little (if any) understanding of the phenomenological dimensions of spontaneous male-on-male attacks:

These sort of laws … might be important in regards to actual gang activity and gang violence. I mean, unless we are missing context here, these guys weren’t in a gang; they were just a bunch of friends. And it’s obvious that they didn’t intend for anyone to die, so they shouldn’t be charged with murder. I mean, that seems pretty obvious to me (Spencer, FG4).

At the most basic level, many focus group members were uncomfortable with the idea that a person could be liable for a crime they did not physically commit. This is where the distinction between assisting or encouraging the offence and assisting or encouraging the offence *with the intention to do so* impressed most heavily. In a different vein, but to similar effect, focus group members were concerned that the meaning of particular actions or words, when examined forensically, could be (mis)interpreted with profound cynicism, given the obligation of the State to vindicate the dignity of murder victims and to express the community’s disapproval of that offending. The use of a simple phrase, like ‘let’s get them’ provides a convenient example. In this case, it was upon those three simple words that the whole joint enterprise case relied, because for the Crown to establish a plan to kill – or foresight of the possibility of death – the prosecution needs those three words to carry a very clear and specific meaning. As such, ‘Let’s get them’ is tantamount to ‘let’s all go and commit murder’ or ‘let’s all go and do GBH’. For members of focus groups, this proved a bridge too far:

My personal view is that the murderer is the person who commits the act, not the people that are there for whatever reason they’re there. Because otherwise, how many of us could be convicted of crimes? It’s that slippery slope thing (Leanne, FG1).

There is clearly responsibility on the part of the person who eggs on another person to behave in a way that is inappropriate. But whether it’s the same responsibility as the person who actually does the deed? I don’t agree (Anthony, FG5).

In this context, most participants argued that Max – and only Max – was responsible for the victim’s death:

As much as I don’t like Robbie from what I’ve seen of him – as a human being I don’t like him – but I just don’t feel that … you can find people guilty for being assholes kinda thing. I feel he’s definitely guilty of assaulting the other guy with the plank of wood. I just think the knife thing is, like, it’s just a sad set of circumstances that Max’s ended up in (Bec, FG7).

### In youth we learn: implications of developmental immaturity

Previous research has shown that young people, aged 14–24 years are overrepresented in multi-defendant prosecutions under joint enterprise (Mills et al., 2022). For this reason, the scenario provided to focus group members involved persons under the age of 25 years. There was a broad consensus about the signature qualities of youth, with community members using words such as ‘tunnel vision’, ‘hot-headed’, ‘emotional’, ‘vulnerable’, and ‘susceptible to peer influence’. Much of the criticism directed at EJCE, centred on its disproportionate impact on young people, who, by their very nature, were recognised to exhibit a transient but nonetheless spectacular inability to forward plan, evaluate competing decisions, and weigh the consequences of their decisions (as an adult would). Effectively, community members understood adolescents to be sensitive to the perceived and generally short-term rewards (peer group inclusion, bravado, excitement) of engaging in risky behaviour, while concurrently having less efficient control networks. This was seen to lead to an increased risk of poor decision-making, particularly in situations where immediate rewards are highly valued:

They certainly went there in a hyped-up, revved-up, aggro sort of state. And young people, particularly young males, just don’t – *can’t* – see where these things are going to go. … So a legal definition of [murder] might be very valid … but it doesn’t accommodate that sort of irrationality (Ted, FG10).

I’ve worked with a lot of sixteen-year-old boys … and that’s pretty standard kind of thinking, you know. Concerningly, not a lot goes on in those brains, from my point of view, sometimes (Michael, FG8).

At the same time, young people were recognised to ‘have limited control over their own environment and lack the ability to extricate themselves from horrific, crime-producing settings’ (*Miller v Alabama*, 2012, 132 S. Ct at 2464). Some community members specifically spoke to the likelihood that young people allowed themselves to become involved in a confrontation out of a sense of bravado but were quickly out of their depth and were indeed in real physical danger. In these situations, secondary offenders were seen to be limited in their ability to undo or neutralise the effect of their previous engagement to avoid liability, as common law requires.

Just because he went there and yelled at a man as a fight broke out – we don’t know that he’s not having his own head smashed on the ground (Tanya, FG2).

I can’t see any 16-year-old with his 18-year-old, 19-year-old mates – he is not going to be the dominant male – that says, ‘Hang on, guys: let’s chill out. Let’s all go home.’ You are the youngest one in that group. You’re not the leader; you’re the follower (Elise, FG7).

### [In]equality before the law: the impact of marginal status

As stated at the outset, the academic literature is clear that young males from minority ethnic communities, particularly the Black community, are consistently overrepresented in multi-defendant prosecutions and convictions for homicide (Mills et al., 2022). The extent to which doctrines of complicity disproportionately impact social groups that carry the weight of current, historic, and personal disadvantage was also a significant issue for community members. Many community members expressed, with some frustration, that the criminal law often dismissed the impacts of entrenched disadvantage and intergenerational trauma, impacts which they believed were significant:

It’s the postcode you’re born in basically. What if Robbie turns out to be an Aboriginal man? Is that going to make a difference? I reckon it does. And this is my advice from 35 years spent up in the [Northern] Territory; that’s just what I see: 85% of the people in gaol are Aboriginal or Torres Strait Islander. And we don’t know what kind of party this was, but if we sort of put the lens on that this is a nice party in Burnside [i.e., an upmarket suburb] versus something up in the APY Lands, it’s different (Jason, FG6).

Several focus group participants also queried the extent to which common law doctrines of complicity relied on outmoded or plainly incorrect assumptions about human behaviour and the capacity of every individual to make ‘good’ choices at critical moments. As described by the community members below, to focus on each situation simply as the sum total of an individual’s bad choices was to ignore the longer and wider view of the historical, social, and cultural landscape. No one, for example, gets to choose their family (see discussion in Halsey & Deegan, 2015, p. 166). For participants, there was a pressing need to remember that all choices are made in a particular context, a particular place, and a particular time. In other words, *who*, *where*, and *when* you are plays a role in shaping the range of choices open to you. For example:

It just seems so out of date and out of touch with society in general. Considering how much evidence there is now around like psychology, socio-economic demographics, cultural things and all sorts. And it’s a little bit sad, I guess for lack of a better word, was that it’s just this all-encompassing broom that everyone gets swept up in and doesn’t consider how much more we know, generally, since that law came in. It should be based on evidence (Nina, FG8).

Others were of the view that the criminal justice system does not do enough to create social awareness of risky situations (educating potential offenders), thereby undermining any suggestion that such complex legal principles could possibly have a deterrent effect:

A 16-year-old? Do you think he understands the minutiae of this sort of thing? (Ted, FG10).

If none of us know these legal things, then this cannot be seen as preventative at all (Maria, FG4).

This [doctrine] is made by people who are educated and have no perspective of what it looks like from someone who may not have reached that level of education or has access to that level of education (Jimmy, FG8).

In this context, focus group participants called for more education in Australia to ensure that all young males were aware of secondary liability – especially in light of the profound and longstanding effects a conviction for murder automatically attracts.

### Brothers in arms: the minority view

A small number of focus group members felt strongly that those who were minded to participate in group violence should inevitably be obliged to accept responsibility, not only for their own actions but also for the actions of other group members when crimes such as these occur:

We’re not talking about going down to the shops and stealing a lolly. We’re talking about an act that’s aggressive and has cost a person their life. I don’t care where you live or who you are. At 19, unless … you’ve been living under a rock, you know what the consequences are for your actions (Lawrence, FG2).

I’m taking in everyone’s discussion of, you know, you almost feel sorry for them because something much worse than what they thought would happen, happened. But once you’re engaging in pretty serious crime or just crime in general, it kind of throws away a little bit for me personally, that consideration (Jay, FG6).

He was with his mates and his mates were his weapons … the three of them. Three of them going to deal with one person at a party. … It’s a bit like jumping in a car when you’ve been drinking. You know, you have no intentions of having an accident but if you do and you are found to be impaired, then there are consequences (Beth, FG2).

For the very small number participants who maintained a sense that the entire group was guilty of murder, the more legitimate question concerned the issue of an appropriate sentence for each defendant. As detailed below, these focus group members were almost universally agreed that a sliding scale of sentences was the correct way to distinguish between different levels of culpability, thereby rejecting the idea of a mandatory sentence. For example:

I don’t really like this idea that … Pete’s almost going to, not scot-free, but he’s going to be not involved [in the murder]. … I’m comfortable with maybe the way that ‘Yes, they are guilty of murder’, but I’m also comfortable with this idea that not everyone who is guilty of murder needs to get the same sentence (Peter, FG6).

### Punishment and society

Leaving aside the issue of whether secondary offenders were morally blameworthy for *causing the victim’s death*, it was very clear that, in the eyes of the public, ‘some defendants who are convicted of murder do not deserve anything like the same degree of moral censure and punishment as others’ (Mitchell & Roberts, 2012, p. 7). To this extent, the ballpark non-parole periods the public attributed to secondary offenders convicted of murder in this scenario often depart significantly from the 20-year mandatory minimum in South Australia. For example:

I don’t believe that Robbie has gone to the extreme to encourage and make sure the murder happened. But it’s still a very serious event. I’d still give a quite serious 8 to 10 year sentence (Craig, FG5).

We can say, ‘yes, they’re young, they’re silly’, okay, but that’s not an excuse for what happened. I would give Pete 1 or 2 years but, on the understanding he is going to receive help for him to be able to deal with situations like that in future (Carrie, FG11).

Absolutely no, no prison time whatsoever for Sam. That’s definitely off the table. I cannot see *any* conceivable way you could make a case for putting him in prison in this scenario and specifically, even a criminal conviction because of what it will do to his life. You might get him to do some community service or something to make it very clear to him that it’s not acceptable behaviour (Ted, FG10).

Focus group members wanted sentences to reflect the contribution of each defendant and their unique circumstances to the events that occurred. This view was shared among participants whether they believed a murder conviction was justified or not. Irrespective of mandatory sentencing, of course, courts must weigh a wide range of offence and offender-related factors in order to determine the severity of the sentence (Warner et al., 2017). Mapping neatly onto known factors that reflect a higher or lower level of culpability, members of the public cited the use of a weapon, the type of weapon, intention to cause harm, justice for the victim’s family, and pre-meditation as aggravating factors. Age, background, perceived spontaneity of the crime, conduct immediately after the incident (i.e. calling an ambulance), and remorse were cited as mitigating factors. As has been observed in literature, EJCE cases often involve groups of young men who are minded to enter an affray with another group of males and to assault the members of that group (Deegan, 2021; Richards & McNamara, 2018). They are the cases where mutual aggression between two opposing groups results in a situation ‘where what is going on is a fight in which the fighters have willingly joined in, whether to carry out or settle a quarrel, or for some other reason’ (*R v Nguyen*, 1995, at 407). Here community members talked about defendants who had been bullied, harassed, or assaulted by the victim as a means to understand – though not justify – their subsequent criminal actions. This led to calls to recognise the active role that victims can play in their own demise, as willing combatants in, or instigators of, the physical attack that provoked the lethal response:

All parties are involved in the brawling side of it. It’s not like they were standing around drinking cups of tea and minding their own business and getting assaulted. They’ve all got a bit of a role to play. So there’s a mitigation for me in that the fact that there was a brawl rather than a casual [one-sided] assault (Mary, FG8).

Relatedly, there was a resounding view that prisons were universities of crime and, as such, should be used only as a last resort for those who were too dangerous to supervise in the community. In this sense, community members argued that community-based sanctions should be used to address the unacceptable behaviour of peripheral actors without exposing them to the problems of the prison community. Again, members of the public circled back to the way in which doctrines of complicity, particularly EJCE, seemed to over-criminalise secondary parties:

We can’t fill our gaols up with everybody that goes along with a mate in a hot-headed situation. [Pete and Sam] would be a community service and an education programme about behaviour. I mean, you cannot fill the gaols with *potential* people all the time. It’s ridiculous. We don’t have the gaol system (Louise, FG10).

I feel like sentencing them to lots of years of community service. I actually think sometimes that going out and helping people who’ve been brain damaged in fights and stuff like that and having to help be their carers is sometimes a lot more useful than a slap on the wrist or spending six months in gaol. Gaol, they often find more of their mates in there and they certainly learn a lot more criminal things when they’re in there (Yvette, FG6).

For other participants, there was another reason to be concerned about the imposition of lengthy sentences on secondary offenders, and the reason avoided debate on whether the offenders deserved a second chance to make good. Namely, allowing participants to serve their sentence in the community, where possible, was important because it made economic sense. As observed by the participants below:

It’s costing us money. Even in just a pure practical sense, it’s costing us money every time someone goes in gaol when it’s a damn lot cheaper to have them out. We need to look more at prevention rather than punishment (Yvette, FG6).

The shock jocks say, ‘Lock them up, throw the keys away et cetera.’ But the thing is, everything costs money and … they come out worse off in many ways. There’s a cost to incarcerating people. I mean, about $35,000 per prisoner per year or something like that. Multiply that by the X amount of people in prison. Who’s paying for that, you know? Us tax folk (Jason, FG6).

As Jason infers, incarcerating a life prisoner occurs at substantively great cost to the taxpayer. As scholars note, ‘locking someone up is roughly 20–30 times more expensive to per day than supervising someone in the community’ (Halsey & Deegan, 2015, p. 197). At a time when Australia and other high-income nations are facing a cost of living crisis, which risks individuals being unable to afford essentials such as heat, food, and clothing, justice reinvestment – or the ‘redirection of money from prisons to fund and rebuild human resources and physical infrastructure in areas most affected by high levels of incarceration’ (Australian Law Reform Commission, 2018, at 4.7) has never been more timely. I do not dwell on it here, but draw attention to preventive measures (education, conflict resolution training, knife crime awareness, efforts to tackle hegemonic masculinity, etc.) that go beyond fairly cursory, if well-intentioned, tough-on-crime responses.^14^

### Mandatory sentencing for murder

Taken together, the data emerging from surveys and focus groups were incompatible with current sentencing arrangements for mandatory murder sentencing in Australia. In fact, irrespective of their level of punitiveness across the scenario, most focus group participants were very clear about their distrust of mandatory sentencing regimes, for murder or any other offence. In this sense, mandatory sentencing was reported to *decrease* rather than *increase* public confidence in the criminal justice system:

I’m comfortable with the finding of murder for everybody because I think that the conduct *is* murder. What frustrates me about the whole situation, what I don’t like, is things like mandatory minimums. And it comes back to this idea. I think a lot of us have really diverse ideas about what things should be considered. We all think people should be treated differently. And I think for something like murder, it’s not super common. And so, I think you can have a case-by-case approach (Michael, FG6).

For the majority of community members, the potential injustice created by mandatory minimums for murder was at its highest, as it concerned children convicted as secondary parties and sentenced as adults (as per s 29(4)(a)(b) of the *Young Offenders Act 1993* (SA) and s 7(a) of the *Youth Justice Act 1992* (Qld)).

The youth get the same minimum sentencing? This is ridiculous. Holding an underdeveloped brain to the same standard with such life-altering consequences (Heidi, FG8).

I don’t get the concept of trying … someone who’s not an adult *as an adult*. Seems like an abstract concept: like they’re either an adult or they’re not. I don’t really care what they’ve done. Yeah. It’s terrifying (James, FG6).

In the end, though, the potential benefits of mandatory sentences for murder really came down to a few short questions. The first was obviously whether it made sense to start all offenders from the same baseline:

Judging people, like these 18-year-olds, with people like Bradley John Murdoch, which was a different beast of a murder, that doesn’t seem like justice or it doesn’t seem fairness. And the idea of a fair go: Australia is built on the idea of a fair go (Leanne, FG1).

The second was whether it was appropriate for Australian parliaments to be intervening in sentencing practices given the strength of, and focus group participants’ belief in, judicial discretion and independence:

Mandatory sentencing, to me, is taking away the responsibility and public view [i.e. the accountability] of the judges. You’re protecting them: ‘Oh, I can’t do anything else. It’s a mandatory sentence; that’s what I have to give. So, I’m not going to take all those things into consideration.’ And to me that’s not right (Connie, FG11).

The third question was similar but different: In the absence of (accurate) public knowledge about crime and justice matters, like the various pathways to a murder conviction, whose interests were actually being served by the implementation of harsh sentences?

For me, this is where law meets politics. Clearly there are votes to be had in crime control and controlling the crime rate. And generally speaking, the tougher you are on crime, the more chance you have of being elected. I don’t know why there isn’t the separation from that. I don’t quite know technically how to do that, but it seems to me that that’s what’s leading to the kind of scenario we’ve heard today. All of us, I think, are shocked. Certainly I am by that, by those outcomes (Anthony, FG5).

As Anthony suggests, evidence abounds that the grip of ‘penal punitiveness on mass opinion and electoral politics is … such that it is ‘a stance that no serious politician can safely disavow’ (Jennings et al., 2017, p. 463; see also Bottoms, 1995; Garland, 2001). Characterised by harsher sentencing, the increased use of imprisonment, more restrictive community orders, and other innovative and experimental forms of crime control (e.g. extended supervision orders), populist punitive culture is often correlated with rising fear of crime and tendency to (erroneously) believe that violent crime is on the rise (Deegan & Jane, 2025; Gelb, 2009). A small number of focus group participants continued to advocate for mandatory sentencing in murder cases irrespective of the circumstances of the crime and against the weight of opposing views within the room. Something of this dynamic is captured in the following exchange:

Harold: Mandatory sentencing: it’s really the government using the courts, playing it tough. Usually before election time, ‘law and order’, so we’re going to bring in mandatory sentencing: ‘If you commit a crime, you’ll get 20 years, 10 years’, whatever that crime is. That’s all it is. It’s appeasing the public’s revenge; that’s all it is. Nothing to do with rehabilitation; it’s just punishment. It’s just more votes for the politicians and to appease the public.

Facilitator: Nathan?

Nathan: But isn’t that what it’s supposed to do? The law is there to reflect what the public wants at the time and to say, ‘Well the community thinks that’s the wrong thing.’ I think Sam was probably hard done by, but he’s still got a dead kid to account for. We’ve talked a lot about these people and their backgrounds but not much about the victim. … He’s still dead. These people aren’t. So we’re valuing these four people’s lives above the dead person, and that always concerns me. We’re saying, ‘Your life’s worth *this*; this other person is worth *that*’ (FG11).

### Concluding remarks

In the context of JCE and constructive murder, it is often said that ‘society has little sympathy for persons who kill in the course of the commission of a serious crime’ (NSW Law Reform Commission, 2010, at [5.57]). This has led some commentators to remark that the public ‘apparently does not share some academics’ views that it is unjust to convict a person of murder because he/she, or an accomplice, unintentionally killed somebody while committing a very serious offence’ (Dyer, 2017, p. 246). Against such a backdrop, this South Australian study explored public knowledge and perceptions of criminal liability and sentencing in the context of murder offending. It was designed to provide a deeper understanding of what the community wants from the criminal justice system and to produce evidence and knowledge relevant to the reform of law about whether some offenders, convicted of murder under EJCE and/or other forms of extended criminal liability, will not merit the conviction or the mandatory sentence for murder but rather some lesser penalty.

The findings in this study reflect, to a large extent, the wider literature that identifies how important it is to hear from the public directly, as well as the key factors that are required to enhance, and not undermine, confidence in the criminal justice system. These findings outline how intention to kill and having some sense of an individual’s active participation in the killing is important to most community members. The majority focused attention on the brutality of the lethal act, the ability of secondary parties to extricate themselves from a difficult situation, the age of the individuals concerned, and the gravity of the original offence (or plan). For many community members, a murder conviction simply ‘didn’t make sense’. However, a small number of people were unhappy about the idea that defendants could escape liability for murder because they had qualms or wished they had not become involved when things went awry. Or, as one focus group participant surmised, ‘because something much worse than what they thought would happen, *happened*’ (Michael, FG6).

Most participants in this sample criticised doctrines of extended criminal liability, particularly EJCE, for its dragnet effect and its apparent disproportionate impact on some of the most disadvantaged and vulnerable members of the community. This included children and young people, for whom the executive functions have not yet fully developed and are appreciably different to adults in how their strong emotional responses are able to be controlled. Of course, the tendency for juveniles to commit crimes in groups and their susceptibility to doctrines of extended liability – such as joint enterprise in the United Kingdom and Canada and the Felony Murder Rule in the United States – and their vulnerability once transferred to the adult criminal justice system is an international issue (see Garberg & Libkuman, 2009; Hulley & Young, 2022). With a particular focus on the neurodevelopmental aspects of a socially disadvantaged group, members of the public indicated a preference for jurisprudential response to spontaneous group violence to be shifted towards issues of culpability and responsibility and away from the unhelpful notions of foresight and (purported) gang membership. Community members were particularly concerned to learn that the same life sentence and 20-year minimum term of imprisonment that apply to adults are also visited on children and young people convicted of murder as secondary parties.

Scholars argue that understanding knowledge or perceptions of crime is critical to understanding opinion surrounding sentencing (e.g. Mitchell & Roberts, 2012; Roberts et al., 2012; Warner & Davis, 2012). Indeed, if sentencing practices are designed to reflect public sentiment, there is some expectation that lay people have knowledge of these practices. Given that most community members were not aware of legal doctrines of complicity, especially EJCE, participants argued that more investment is needed to ensure that all citizens know what the law is and what would likely happen when events like these occur. They argued that the reform of law to protect vulnerable members of the community and a focus on violence prevention are warranted. For example:

I’m going to write to the Attorney General about this (Leanne, FG1).

I came in here thinking I was fairly tough on sentencing … but I am not as tough as the law obviously is. I didn’t realise everyone would get life (Don, FG1).

Following the case of *R v Jogee* [2016] UKSC 8, the United Kingdom has, in theory at least, abandoned EJCE, or ‘parasitic accessory liability’, on the grounds it is ‘illegitimate … to treat foresight as an inevitable yardstick of common purpose’. But the true challenge for that jurisdiction, it would seem, is to translate the ruling of the Supreme Court in *Jogee* into fewer EJCE prosecutions and fewer crushing sentences (Hulley & Young, 2025; Mills et al., 2022).

Given the clear commitment of prosecutorial agencies to persist with EJCE, legislative change to mandatory sentencing regimes is critical to moderate against the disproportionate and discriminatory impact EJCE has against the most vulnerable populations. As I have argued in this article, mandatory sentencing laws – especially when used in these cases – run counter to public safety, fiscal responsibility, and justice.

Alternatively, and most pragmatically, the present research calls for the scope of mandatory sentencing laws to be significantly reduced in their application to JCE homicide cases. In the study location of South Australia, for example, under s 48(3) of the *Sentencing Act 2017* (SA), limited scope exists for a court to depart from the mandatory minimum non-parole period if satisfied that ‘special reasons’ exist. Special reasons include whether the offence was committed in circumstances in which the victim’s conduct substantially mitigated the offender’s conduct and/or in light of the circumstances/consequences of their guilty plea and cooperation with the police investigation. However, it has been said that the gravity of the offence of murder will generally render the mandatory minimum sentence inevitable in order to achieve the objects prescribed by statute (Deegan, 2021). It is therefore highly unlikely that a conviction under EJCE could, at present, qualify as a ‘special reason’ to depart from the mandatory minimum sentence. Given that so many of the community members in this study reported unease regarding current legal arrangements for murder, an express provision exempting EJCE cases from mandatory sentencing regimes is necessary. The current work indicates that this is not only palatable to the community but actually desired by it. This would go some way to fostering a judicial system that not only promotes fairness and equality before the law but also respects and values public opinion and demonstrates this respect in all that they do.

## Footnotes

1 *Criminal Justice Act 2003* (UK) s269: A life sentence is defined (by section 277) as meaning a sentence of imprisonment for life, which is imposed on those convicted of murder who are 21 or more years of age, a sentence of custody for life, which is for those aged 18, 19, or 20 years of age, and a sentence of detention during Her Majesty’s Pleasure, which is for those under 18 years of age. See also *Sentencing Act 2020* (UK). Schedule 21: Determination of minimum term in relation to mandatory life sentence for murder.

2 See Appendix 1 for a summary of penalties for murder in each Australian state and territory.

3 *Criminal Code* (R.S.C., 1985, c. C-46): Punishment for murder, s235 (1) Every one who commits first degree murder or second degree murder is guilty of an indictable offence and shall be sentenced to imprisonment for life. Marginal note: Minimum punishment (2) For the purposes of Part XXIII, the sentence of imprisonment for life prescribed by this section is a minimum punishment.

4 *Young Offenders Act 1993* (SA) s29(4).

5 *Youth Justice Act 1992* (QLD) s7(a); see also ‘Making Queensland Safer Laws to restore community safety’, https://statements.qld.gov.au/statements/101648.

6 In the context of a wider study of public opinion and sentencing for murder, Mitchell and Roberts (2010, reprinted in 2012), ‘briefly took the opportunity to gauge public opinion on joint enterprise murder’ (p. 4) in the United Kingdom. This was not a central aim of the study and only tested public preference in relation to a murder or manslaughter conviction in these cases. The study did not interrogate the rationale for the public’s preferences or deal with the question of sentence.

7 The case of *R v Stokes and Difford* (1990 51 A Crim R 25) requires the prosecution to prove beyond a reasonable doubt that an accessory knew or was aware of the principal’s intention to do the act that caused the harm and that the act was done maliciously.

8 In South Australia, where this was study was conducted, s12A of the *Criminal Law Consolidation Act 1935* (SA) is the statutory replacement for the common-law felony-murder rule.

9 In South Australia, for example, a serious crime is defined as a major indicatable offence, punishable by 10 years’ imprisonment or more (s12A *Criminal Law Consolidation Act 1935* (SA)).

10 The Australian High Court was invited to reconsider the position, see *Miller v The Queen; Smith v The Queen; Presley v Director of Public Prosecutions* (SA) 2016 HCA 30. The principles in *McAuliffe v The Queen* were upheld.

11 Ethics approval for this project was granted by the Flinders University Human Research Ethics Committee, Project ref #5228.

12 Results of phase one are published: Deegan and Jane (2025).

13 *R v Barlow* (1997) 188 CLR 1 outlines the instances where common law is applicable to a codified offence, for example, in the case of ambiguity. The judgment also highlights the need to strive for uniformity between code and common law states.

14 See, for example, work by the Ben Kinsella Trust; The Sammy D Foundation; Alliance of Sport in Criminal Justice.

## Legislation

*Criminal Justice Act 2003 (UK).*

*Sentencing Act 2020 (UK).*

*Criminal Code* (R.S.C., 1985, c. C-46).

*Young Offenders Act 1993* (SA).

*Youth Justice Act 1992* (Qld).

*Sentencing Act 2017* (SA).

*Criminal Law Consolidation Act 1935* (SA)

## Cases

*Chan Wing-Sui v The Queen* [1985] AC 168

*McAuliffe v The Queen* (1995) 183 CLR 108.

*Miller v The Queen; Smith v The Queen; Presley v The Director of Public Prosecutions* [2016] HCA 30.

*Mitchell v The King* [2023] HCA 5.

*The Queen v Stokes and Difford* (1990) 51 A Crim R 25.

*Giorgianni v The Queen* (1985) 156 CLR 473.

*Huynh v The Queen* (2013) 87 ALJR 434 at [38].

*R v Sully* (2012) 112 SASR 157 at 178.

*Arulthilakan v The Queen* (2003) 78 ALJR HCA 74.

*R v Ryan and Walker* (1966) VR 553.

*R v Jogee* [2016] UKSC 8.

*R v Nguyen* (1995) 36 NSWLR 397.

*Stuart v The Queen (*1974) 134 CLR 426.

## Appendix

**Table:**

Australian State/Territory	Item no	Offence	Non-parole period
Northern Territory	*Criminal Code Act 1983* (NT)	**Section157 Punishment for murder and conspiracy to murder** (1) A person who is guilty of the offence of murder is liable to imprisonment for life. (2) **The penalty mentioned in subsection (1) is mandatory**.	Where a court (the sentencing court) sentences an offender to be imprisoned for life for the offence of murder, the court **must fix** under section 53(1): A standard non-parole period of 20 years; If any of the circumstances in subsection (3) apply – a non-parole period of 25 years. The standard non-parole period of 20 years referred to in subsection (1)(a) represents the non-parole period for an offence in the middle of the range of objective seriousness for offences to which the standard non-parole period applies - *Sentencing Act 1995* (NT) section 53A
South Australia	*Criminal Law Consolidation Act 1935* (SA)	**Section 11 Murder** Any person who commits murder shall be guilty of an offence and **shall be imprisonment for life**.	If fixing a non-parole period in respect of a person sentenced to life imprisonment for an offence of murder, **the mandatory minimum non-parole period** prescribed in respect of the offence is 20 years. - *Sentencing Act 2017* (SA) s 47(5)(b)
Queensland	*Criminal Code Act* 1899 (QLD)	**Section 305 Punishment of Murder** Any person who commits the crime of murder is **liable to imprisonment for life, which can not be mitigated or varied** under this *Code or any other law or is liable to an indefinite sentence under part 10 of the Penalties and Sentences Act 1992.*	For subsection (1)(a), the period of time the offender **must have served** is – a) for an offender whose nominal sentence is life imprisonment for an offence of murder if the Criminal Code, section 305(2) applies on sentence – 30 years; or if the Criminal Code, section 305 (4) applies on sentence – 25 years; or **otherwise – 20 years**; b) *Penalties and Sentences Act 1992* (QLD) Part 10 Indefinite Sentences
Victoria	*Crimes Act 1958* (Vic)	**Section 3 (1) Punishment for murder** Notwithstanding any rule of law to the contrary, a person convicted of murder is liable to (a) level 1 imprisonment (life); or (b) imprisonment for such other term as is fixed by the court	**The standard sentence for murder** is – a) 30 years if the court is satisfied that the prosecution has proved beyond reasonable doubt items (i) and (ii). b) In any other case, 25 years. c) *Crimes Act 1958* (Vic) s 3(2).
Tasmania	*Criminal Code* Act *1924*	**Section 158 Murder** any person who commits murder is guilty of a crime, and is liable to imprisonment for the term of the person’s natural life or for such other term as the Court determines	Whether a person convicted of murder will have any non-parole period, and the length of that period, is dependent on whether or not they are sentenced to imprisonment for the term of their natural life. The standard statutory non-parole period is one half of the term of imprisonment under s 68 of the *Corrections Act 1997* (Tas). This does not apply to persons sentenced to imprisonment for the term of their natural life. Persons sentenced to imprisonment for the term of their natural life have their eligibility for a non-parole period determined by the sentencing court under s 18(1) of the *Sentencing Act 1997* (Tas). They may or may not have a non-parole period set. Under s 18(2), the court must consider the offence’s nature and circumstances; the antecedents/character of the offender and any other sentence they may be subject to. If a person does not have a non-parole period set then they are not eligible for parole (s 18(4)).
Western Australia	*Criminal Code Act Compilation Act 1914* (WA)	**Section 279 Murder** 4) A person, other than a child, who is guilty of murder **must be sentenced to life imprisonment** unless a) that sentence would be clearly unjust given the circumstances of the offence and the person; and b) the person is unlikely to be a threat to the safety of the community when released from imprisonment, in which case, subject to subsection (5A), the **person is liable to imprisonment for 20 years**.	**Life imprisonment for murder, imposing** A court that sentences an offender to life imprisonment for murder **must either** – Set a minimum period of – at least 15 years, if the offence is committed by an adult offender in the course of conduct that constitutes an aggravated home burglary; or at least 10 years in any other case; or order that the offender must never be released. *Sentencing Act 1995* (WA) – Part 2, Division 1 – Sentencing Principles – item 90(1) **Release from life imprisonment** A prisoner serving a sentence of life imprisonment for murder in respect of which a minimum period has been set under section 90(1)(a) is not to be released before he or she has served the minimum period *Sentencing Act 1995* (WA) – Part 2, Division 1 – Sentencing Principles – item 96(1)(a)
New South Wales	*Crimes Act 1900* (NSW)	**Section 19A** **Punishment for murder** A person who commits the crime of murder is liable to imprisonment for life. A person sentenced to imprisonment for life for the crime of murder is to serve that sentence for the term of the person’s natural life.	**Section 61 Mandatory life sentence for certain offences** **A court is to impose a sentence of imprisonment for life** on a person who is convicted of murder if the court is satisfied that the level of culpability in the commission of the offence is so extreme that the community interest in retribution, punishment, community protection and deterrence can only be met through the imposition of that sentence. **Standard non-parole periods** 1A Murder – where the victim was a public official: 25 years 1B Murder – where the victim was a child under 18 years of age: 25 years 1 – in other cases: 20 years
Australian Capital Territory	*Crimes Act 1900* (ACT)	**Section 12(2) Murder** A person who commits murder is guilty of an offence punishable, on conviction, by imprisonment for life.	***Crimes (Sentencing) Act 2005*(ACT)** Non parole periods are not ordinarily set by the sentencing court for sentences of life imprisonment**(***Crimes (Sentencing) Act 2005* (ACT) s 65(5).
